# Application of a faith-based integration tool to assess mental and physical health interventions

**DOI:** 10.21633/jgpha.7.105

**Published:** 2017

**Authors:** Donna M. Saunders, Jean Leak, Monique E. Carver, Selina A. Smith

**Affiliations:** 1 Guide Community Health Services Inc. Ellicott City, MD; 2 Refreshing Springs Outreach Sykesville, MD; 3 Department of Family Medicine, Medical College of Georgia at Augusta University, Augusta, GA

**Keywords:** faith-based integration and interventions, faith and health studies, religion and spirituality, integrative health

## Abstract

**Background:**

To build on current research involving faith-based interventions (FBIs) for addressing mental and physical health, this study a) reviewed the extent to which relevant publications integrate faith concepts with health and b) initiated analysis of the degree of FBI integration with intervention outcomes.

**Methods:**

Derived from a systematic search of articles published between 2007 and 2017, 36 studies were assessed with a Faith-Based Integration Assessment Tool (FIAT) to quantify faith-health integration. Basic statistical procedures were employed to determine the association of faith-based integration with intervention outcomes.

**Results:**

The assessed studies possessed (on average) moderate, inconsistent integration because of poor use of faith measures, and moderate, inconsistent use of faith practices. Analysis procedures for determining the effect of FBI integration on intervention outcomes were inadequate for formulating practical conclusions.

**Conclusions:**

Regardless of integration, interventions were associated with beneficial outcomes. To determine the link between FBI integration and intervention outcomes, additional analyses are needed.

## INTRODUCTION

Over the last two decades, there has been growing evidence linking faith practices and spirituality to health benefits and clinical outcomes. For example, a review by Koenig (2013), revealed consistent increases in published articles on this topic: 1988–1993, 1976 articles; 1994–1999, 2782 articles; 2000–2005, 4108 articles; and 2006–2011, 5155 articles. Published articles yielded by these reviews - which have included editorial reports and concept papers, epidemiological studies, as well as clinical trials that were primarily conducted in clinical settings such as hospitals and clinics and have included faith measures and practices in the range of risk factors and outcomes involved in these trials - have repeatedly confirmed the health benefits of the ‘faith factor’. Such benefits have included (but are not limited to) longer life, greater life satisfaction and well-being, lowered risk of depression and suicide, improved coping and management of stress, lowered cardiovascular risks, lowered risk and successful recovery from alcohol and other drug addiction, and faster surgical recovery (Larson et al. 1992, Larson, 1994, Matthews et al. 1997, Koenig, 2013).

To offset healthcare costs and increase the reach for influencing the reduction of public health risks in America, interest among healthcare providers, clinical researchers, and public health experts in the incorporation of faith practices and the faith community into the design and implementation of prevention intervention strategies has increased. H. Koenig, psychiatrist and clinical researcher, points out that through the faith community, early detection and prevention of disease, respite care, and facilitation of compliance with treatment can be advanced (Koenig 2013). Also worth noting is that according to a national study (Catanzaro et al., 2007), one third of churches in the United States now have health ministry programs signifying a growing interest for meeting the health needs of parishioners.

Given the emergence of faith-based interventions (FBIs) in public health and the clinical literature addressing health problems ranging from obesity and other cardiac risks to HIV/AIDS prevention to recovery from alcohol and other drug addictions, we sought to investigate the nature and characteristics of FBI integrations and to link them to outcomes. In our preliminary search for published reviews of FBIs, we found only one that probed the extent to which these interventions were truly faith based that is, incorporated some aspect of faith practices or experiences. This study, conducted by Lancaster et al. (2014), however, distinguished only between interventions that were faith-placed (e.g., a non-FBI conducted in a church) and FBIs, and provided no formal procedures for quantifying the extent of FBI integration. Our review, therefore, investigated Lancaster’s topic further by quantifying the extent of FBI integration involved in the faith-based intervention – that is, interventions that were identified as faith-based by virtue of being conducted in a faith community and/or making faith practices and/or beliefs central to the intervention - and by probing how FBI integration was linked to intervention outcomes. The following were our focus: a) What is the extent of research involving FBIs for addressing health concerns – that is, medical, public health, and/or mental health – under empirically controlled conditions?; b) Once found, what is the extent of faith integration, as evaluated by a rating scale designed to assess faith-health integration, that is, the extent to which faith concepts and practices were interwoven into the health intervention?; c) To set the tone for future studies, a secondary question was: In the reviewed empirical studies, to what extent was strength of FBI integration linked *to* or correlated *with* beneficial outcomes?

## METHODS

### Search strategy and inclusion/exclusion criteria

To identify studies meeting the inclusion criteria, we searched Medline, Google Scholar, PsycInfo, PsychArticle, and CINAHL for manuscripts published between January 1, 2007, and February 1, 2017, for those conducted in the US, written in English, and published in peer-reviewed journals. In addition, the advanced title and abstract features of Pubmed, Google Scholar, and EBSCO were used to identify additional articles. The references of relevant articles, including systematic reviews, involving the inclusion of faith and spirituality in health outcomes were also searched. The studies could be randomized controlled trials (RCT’s), quasi-experimental or single-group where there was no control group. Surveys were excluded. Like Lancaster, we sought to identify a representative sample of studies, primarily conducted in the field of public health, whose interventions were identified by investigators as faith based. This being in contrast with Koenig’s work which explores the entire domain of published articles, be they completed interventions, survey studies, position papers, etc., that involve the use of faith as a health-related factor, whether faith-based or not. Our resulting sample, like Lancaster, was expected to be relatively small compared to Koenig. Further, in hopes of providing examples of fully integrated FBI’s in our study, we included systematic reviews of religiously based interventions in clinical research involving hospital based chaplaincy services, pastoral counseling and other religiously based clinical services as a source for identifying FBI’s.

Key words, collaboratively derived by the review team, were: *faith (including church, religion, spirituality) AND intervention (including clinical trial/studies, randomized controlled trial) AND health/mental health (including health education/promotion, preventive/public/community health/mental health)*. The review team consisted of a) a clinical psychologist with a background in researching the link between faith and the promotion of mental and physical health; b) a public health researcher with experience in community-based participatory research that focused on health disparities and cancer control and prevention; c) a registered nurse and clinical pastoral counselor with clinical experience emphasizing women’s wellness, the integration of faith in clinical practice, and health equity; and d) a clinical research professional with experience in monitoring biopharmaceutical clinical research trials and with a background in quality assurance, compliance, institutional review, and subject safety.

**Figure 1:**
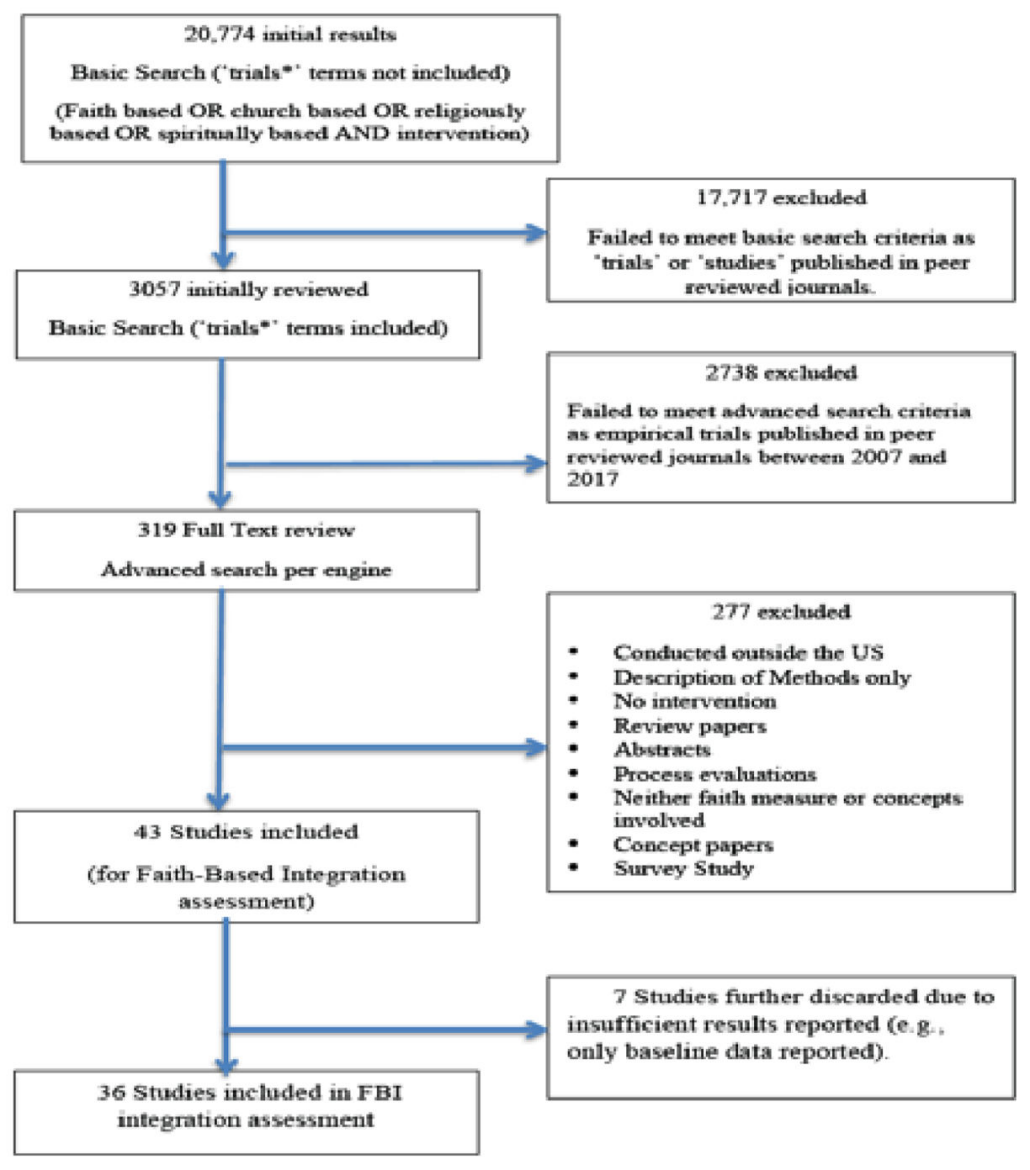
Flow diagram of the literature search

Once identified by the team, articles were assigned in equal portions to reviewers to probe the content in more detail. Consequently, team members ensured that each article met the inclusion criteria and, using a quantitative assessment tool, determined the extent to which faith concepts and practices were integrated into the health intervention. The inclusion/exclusion strategy is summarized in Figure 1.

### Assessment of Faith-Based Integration

The Saunders-Leak Faith-Based Integration Assessment Tool (SL-FIAT) (2017) operationalized a fully integrated FBI – that is, one in which the intervention’s target is, when quantitatively assessed, fully faith-based - as one that: a) gave equal or nearly equal time in its session topics (e.g., prayer, reading and teaching of sacred text, and inspirational music) to faith practices as given to the health topic; and b) utilized four or more of the dimensions of faith experience and practices recognized in the faith-health literature (Larson et al., 1992; Koenig, 2013). This method for operationalizing program targets and measuring an organization’s progress toward achieving program intervention targets along a quantified continuum is utilized in organizational and social psychology (O’Conner, 2002) and makes use of Prochaska and DiClemente’s 5 stages of change (1982). Using this approach, the identified studies were assessed along a continuum of 1–10, where FBI scores of 1–2 reflect no integration, 3–4 reflect low integration, 5–6 reflect moderate or inconsistent integration, 7–8 reflect high integration, and 9–10 reflect full or achieved integration, the derived FBI integration score reflected the sum of the Faith Practices (FP) score and the Faith Measures (FM) score (Table 1).

### FP & FM Scoring

FP scoring ranged from 1–5, where a score of 5 reflected the full integration described above (e.g., time devoted to scriptural teaching vs. time devoted to teaching about diabetes is equal or close to equal). FM scoring, also ranging from 1–5, reflected the amount of faith measures according the criterion for full integration stated above (e.g., four or more faith measures used) (Table 2). Scorable measures were derived from a list reported in the literature (Larson et al, 1992; Koenig, 2013).

### SL-FIAT Reliability Procedures

Reliability of the tool was tested by use of an inter-rater procedure. Initially, the entire team used the tool to rate five studies (Chang et al., 2007, Koenig et al., 2015, Koszycki et al., 2014, Petry et al., 2008, Schoenthaler et al., 2015) that were randomly selected from the 36 studies, revealing strong inter-rater agreement for three of the five studies. After discussing the variation of the remaining two (e.g., how scores were derived) and reviewing methods for extracting faith practices and faith measures from articles, each team member independently re-rated the two outlier studies (Chang et al., 2007, Koszycki et al., 2014). Results of the re-rating were unanimous, suggesting strong inter-rater reliability of the tool.

To examine results of this review, basic statistical analyses, including measures of frequency, central tendency, percentage data collection, and, if conducive, correlational procedures were performed to address the research questions.

## RESULTS

### Sample Size, Study Design, Setting, and Population

Sample sizes in the 36 studies ranged from 2 to 1033 participants. Randomized controlled trials (RCTs), pre-test/post-test (P/P), and quasi-experimental, self-identified studies were included in the analyses. RCT design was confirmed by random allocation to a control group or at least one intervention group. Of the studies assessed, 80% were RCTs. P/P was determined by the availability of before and after intervention data; five (14%) studies had this design. Two (6%) were observational studies. The location of interventions in the sample was defined as church, clinic (hospital, outpatient), or other community setting. Most studies were conducted in churches (46%), followed by community (26%), and clinics (25%). Both men and women were included in most studies (71%), eight targeted only women, one included men only, and one included children as participants (Table 3).

### Included vs. Excluded Studies

The initial search of basic terms (e.g., faith based and interventions) yielded 20,774 results, of which 17,717 were excluded because they were not empirical trials and/or were not published in peer-reviewed journals (N=3057). An advanced search using the same terms yielded 319 studies, for which their abstracts and full texts were reviewed to determine what publications would be assessed by the team using the S-L FIAT. The resulting N for the included studies was 36 (Figure 1).

Of the studies, 27 (75%) used one or more faith measures (e.g., denomination, frequency of religious attendance, extent of religious beliefs), and 27 (75%) incorporated use of faith/spiritual practices in the interventions, with the proportion of time devoted to faith practices falling within ranges from 10–25% to 40–50%. Regarding use of faith measures and proportion of time involving use of faith practices in the interventions, most of the studies incorporated both at least at a minimum level.

Five of the 36 studies included neither faith measures nor faith practices. These were identified as faith-placed interventions, as they made use only of the faith community as their base of operation, making no apparent attempt to incorporate faith practices or measures into them (Table 3).

### FBI Integration, FP, and FM Score Distributions

The mean FBI integration score was 5.75, placing the degree of faith-health integration of these studies at the moderate or inconsistent level. The mode or most frequent categorical score also fell within the moderate integration category, accounting for 11 of the 36 studies (31%)

The average score for faith practices (FP) was 3.17, indicating moderately low use of FPs, suggesting no more than 30% of intervention time being devoted, with use of FPs being either inconsistent or unscheduled. The studies that fell within the moderately high-to-high use of faith practices (N=18) tended to consist of outlined curricula that designated faith-based practices and topics to specific sessions or made faith practices (e.g., daily reading of sacred text) central to the intervention. The larger proportion of studies with high integration involved chaplaincy and pastoral counseling service disciplines or 12-step programs as treatment interventions. These disciplines were recognized for making faith practices part of treatment.

The mean faith measure (FM) score for the studies was 2.58, suggesting relatively weak or low use of faith measures. Of the 36 studies, 22 (61%) fell at or below a score of 2 for FM, indicating the presence of no more than one of the many measures of faith/spirituality reported in the faith-health literature. Eleven studies (31%) yielded moderately high-to-high use of faith measures, using three or more faith/spirituality measures. Such studies were well planned and reflected awareness of current research on the faith-health link and the methods used for measuring the multi-dimensions of this construct.

### FBI Integration and Intervention Outcomes

To probe the relationship between FBI integration and outcomes, the outcomes were divided into two categories: a) negative or non-significant intervention outcomes and b) positive or beneficial significant intervention outcomes. Of the 36 studies, 32 (89%) yielded beneficial and significant outcomes; only 4 yielded negative or non-significant outcomes (Allicock et al., 2012, Cowart et al., 2010, Holt et al. 2013, Koenig et al 2015). Results from this approach to analyzing the relationship between faith-based integration and intervention outcomes reveal little for addressing this point.

## DISCUSSION

This review revealed a prevalence of literature involving the topic of faith and a growing use of FBIs in association with public health prevention and education efforts and with mental/physical health treatment. Consistent with Koenig’s work, published articles involving the topic or the inclusion of faith practices, measures, and/or beliefs in the pool of selected dependent and independent variables for survey studies, epidemiological reports, and clinical trials is also prevalent with outcomes confirming a consistent association of faith involvement, including religious attendance, with health benefits (e.g., longer life, faster recovery, decreased risk of suicide and the negative impact of stress/anxiety and depression, lowered cardiovascular risks, etc.).

As with Lancaster, however, when interventions were identified as faith-based where the focus of the study was highlighted as centrally involving faith (e.g., in the title and/or abstract) and, more importantly, involving the faith community, particularly in the public health arena, the number of studies that were completed and fit our criteria was narrowed. This suggests that while faith as a factor in health is increasingly being included in research literature as revealed by the work of Koenig, Larson, and others (evidence found more in the clinical literature and integrative disciplines like chaplaincy and pastoral care/counseling) and found to be beneficial to health, relatively few completed, scientifically rigorous studies have been done on the integration of faith into health promotion, a principle focus of public health.

Having assessed the 36 FBI studies with our integration tool, which yielded a moderate degree of integration (inconsistent use of faith practices and low use of faith measures), we conclude that, as used in the literature, the term ‘faith-based intervention’ refers to a range of health interventions involving low to inconsistent use of faith practices in interventions and, when FBI integration is quantified, weak to non-existent use of faith measures. In the absence of adequate measures of faith/spirituality, it is difficult to establish the relationship or overall impact of this variable for predicting treatment outcomes in FBIs (e.g., determining for whom a truly integrated FBI is an appropriate treatment or good match for reducing identified health risks and what forms of faith expression are useful for achieving behavior change among varying populations at risk). Further, refining of the definition and measurement of integrated FBIs allows comparisons of their usefulness in relation to non-FBIs in reducing health risks.

Since most (all but four) of the studies were linked to beneficial outcomes (likely an indication of publication bias), the results suggest that more procedures are required to determine the nature and characteristics of the link between the extent of FBI integration and treatment outcomes, providing the basis for a follow-up review. However, three of the four studies whose outcomes were negative or non-significant had FBI scores ranging between low to moderate integration due to low to moderately low use of faith practices (Allicock et al., 2012, Cowart et al., 2010, Holt et al. 2013). This suggests that low use of faith practices in FBIs reduces the likelihood of beneficial treatment outcomes. However, additional analyses of FBI outcomes are needed to substantiate this assertion.

The one highly integrated study that yielded a non-significant outcome was conducted in a clinical setting, comparing the effects of religiously based cognitive behavior therapy (RCBT) with cognitive behavior therapy (CBT) on intervention outcome (Koenig et al., 2015). The conclusion was that RCBT is at least as useful as CBT for reducing symptoms of depression resulting from non-psychotic chronic illness. Consequently, for studies such as this, non-significant results support the hypothesis that FBIs are viable alternatives in the treatment of depression among individuals for whom faith is important.

Relative to the FBI integration-outcome link, the results suggest that: a) a distinction should be made between studies with goals to determine if faith-based approaches to health behavior change are better or worse than non-faith-based interventions and studies of non-faith based interventions; b) investigators should examine under what conditions and for whom FBIs are more appropriate for achieving changes in health behavior; and c) procedures should be identified for determining the strength of outcomes based on a set of criteria (e.g., amount of expected health outcomes that are found significant and how much of the faith-based health outcomes are found to be significant), and, based on these criteria, assess the cumulative strength of significance of each study, correlating strength of outcomes with FBI integration scores. Notwithstanding, results of this review suggest that more analyses of the outcomes of these studies are needed to define the link between FBI integration and intervention outcomes. This provides the basis for a follow-up to this review.

## CONCLUSIONS

Future studies should attempt to incorporate a moderately high to high level of faith practices and a high level of faith measures into the design of the interventions. Studies in which faith concepts and practices are integrated into the health intervention should be measured with tools such as the faith-based integration instrument introduced in this article. Finally, such studies should be approached with greater scientific rigor where study limitations that were characteristic of this cohort (e.g., low quality evidence, small sample size) are addressed.

## Figures and Tables

**Table 1:** Score index for FBI score result

**FBI Index**	1–2 No Integration (no attempt to incorporate faith practices or faith measures in the intervention.)	3–4 Low Integration (low or poor attempt to incorporate faith practices and measures into the intervention)	5–6 Moderate Integration (moderate or inconsistent use of faith practices and measures in the intervention)	7–8 High Integration (high proportion of use of faith practices and measures in the intervention)	9–10 Full Integration (Fully integrated use of faith practices and measures in the intervention)

**Table 2:** Faith practice, faith measure score, and faith-based integration score criteria

FAITH PRACTICE (FP)		
Select the rating that best fits the degree to which the intervention includes faith practices (e.g., prayer, application of sacred text/scripture, worship, music, etc.).		FP =
**5**	Large proportion of curriculum devoted to use of faith practices (within 40–50% range or ½ of the curriculum to FP)	

**4**	Moderately high proportion of curriculum devoted to use of faith practices (within 30–40% range)	

**3**	Moderately low or inconsistent, unscheduled proportion of curriculum devoted to use of faith practices (within. 25–30% range)	

**2**	Very low proportion of curriculum devoted to use of faith practices (within 10–25% range)	

**1**	No discernable use of faith practices included in the curriculum OR unknown OR only placed in a faith community (e.g., faith-placed).	

**FAITH MEASURE (FM)**		
Select the rating that best fits the degree to which the intervention used faith measures (e.g., religious attendance, importance of religion, religious coping, religious satisfaction, religious motivation, denomination, religious support, sacredness of the body, etc)		**FM =**

**5**	High/strong use of faith measures (at least 4 dimensions)	

**4**	Moderately high/strong use of faith measures (at least 3)	

**3**	Moderately low or weak use of faith measures (no more than 2)	

**2**	Very Low or weak use of faith measures (no more than 1 measure)	

**1**	No discernable use of faith measures or unknown	

**Table 3:** FBIs on physical and mental health

Study	Sample	Design	Results	Limitations	FBI Score
Allicock et al. (2012)	1033 members (15 churches), African Americans, average age 52 years	Randomized controlled trial, fruit and vegetable nutrition program with pastoral involvement, educational activities, church environmental changes, and peer counseling	No statistically significant difference in daily servings of fruits and vegetables between early intervention group and control group (4.7 vs. 4.4, p=0.38)	Lack of randomization, use of self-reported outcome measures	4
Anderson et al. (2013)	27 Christian African American churches, females 60 years or older, men 70 or older	Cluster randomized controlled trail with wait-list control group, 10-week, 90-minute physical activity with spiritual strategies	Intervention group improvements in muscle strength activity (minutes per week, *z*= −3.269, p=0.001; days per week, *z* = −3.384, p=0.001) and 6-minute walk change scores (*z* = −2.546, p=0.009)	Uncertain generalizability	7
Bay et al. (2008)	170 coronary artery bypass graft surgery patients from 4 hospitals, 30–60 years of age	Randomized controlled trial, hospital chaplain visits, four for intervention group and one with family members	Increased positive religious coping in intervention group, decreased in controls (p=0.023); negative religious coping decreased in intervention group, increased in controls (p=0.046)	Uncertain generalizability	8
Borman et al. (2009)	93 HIV patients, 18–65 years of age	Randomized controlled trial, 5-week, 90-minute group-based mantram/meditation classes	Positive reappraisal coping increased for intervention group (F (1, 45) =17.97, p < 0.01), decreased for controls (F (1, 46) = 3.98, p=0.05) and decreased anger (F (3, 45) =10.12, p <0.01)	Small sample size, uncertain generalizability	7
Bormann et al. (2008)	33 post-traumatic stress disease (PTSD) combat veteran males, 40–76 years of age	Randomized controlled trial, 6-week, 90-minute mantram intervention	Large effect sizes for reductions in PTSD symptom severity (d =−0.72) and psychological distress (d =−0.73) and increased quality of life (d =−0.70)	Small sample size, uncertain generalizability	7
Bowland et al. (2012)	43 female survivors of interpersonal trauma, 55–83 years of age	Randomized controlled trial, 11-session group intervention using spiritual coping resources	Lower depressive symptoms, anxiety, and physical symptoms among intervention (F(4,38)=8.42, p=0.0059; depression F(1,41)=23.66, p<0.0001; and general health (F(1,41=9.47, p=0.0037)	Social desirability bias	9
Breitbart et al. (2012)	120 cancer patients, 25–82 years of age	Randomized controlled trial, 7-week individualized meaning-centered psychotherapy (IMCP)	Greater improvement in interventions vs. controls for spiritual well-being (b=0.39; p<0.001), sense of meaning (b=0.34; p=0.003) and faith (b=0.42; p=0.03), quality of life (b = 0.76; p=0.013), symptom burden (b=−6.56; p< 0.001), and symptom-related distress (b=−0.47; p< 0.001)	Small sample size, unequal treatment allocation	6
Chang et al. (2007)	119 HIV/AIDS patients, average age 46	Randomized controlled trial, 12 week 2x/wk for 20 minutes acupuncture treatments with taped instructions plus music for relax. techniques	Intervention improvements in emotional (p=0.0002), spiritual (p=0.02), physical (p=0.003), and mental health (p=0.0003)	Small sample size, uncertain generalizability	6
Cowart et al. (2010)	2 participants, 18 and 70 years of age	Pre-post test of 12-week nutrition education and exercise fitness intervention	Post-intervention reductions in frying meats (69% vs. 50%)	Small sample size, lack of randomized controlled design, uncertain generalizability	3
de Mamani et al. (2016)	113 schizophrenia caregivers from 66 families, average age 53.7 years	Randomized controlled trial, psycho-education/cognitive behavioral techniques (Psy-Ed) or Psy-Ed plus family focused culturally informed treatment (CIT)	CIT outperformed Psy-Ed in reducing guilt/self-blame (Beta = 397, p <0.05) and caregiver burden (Beta= 2.058, p <0.01); reductions in guilt/self-blame mediated changes observed between treatment type and caregiver burden ((β =0.497, p <0.05)	Uncertain generalizability	4
DiGuiseppi et al. (2014)	51 churches, 7101 members, 60 years of age or older	Randomized controlled cluster trial, 4-wave church-based social marketing program	Intervention churches had a higher mean number (7.0 vs. 0.5; IRR = 11.2 [95%CI: 7.5, 16.8]) of older adult congregants who joined balance classes and were more likely to recall information about preventing falls with balance classes (AOR=6.2; 95% CI: 2.6, 14.8)	Social desirability bias, small sample size, uncertain generalizability	3
DJuric et al. (2009)	31 obese African American breast cancer survivors, women, 18–70 years of age	Randomized controlled trial, 18-month dietary only or dietary and spiritual counseling by phone using Weight Watchers program and professional counselor	Spirituality counseling positively affected spiritual well-being (r=0.599, p=0.026) and dietary quality (change in diet group 0.3 compared to 1.2 in spirituality group, p=0.012)	Small sample size, uncertain generalizability	7
Dodani & Fields (2010)	40 high-risk type II diabetes, African Americans, 18–64 years of age	Feasibility trial, church health minister-delivered 12-week, 1-hour sessions with a 6-month booster	Post-intervention weight change: 48% lost ≥5% of baseline weight; 26% lost ≥7%; and 14% lost >10%	Small sample size, uncertain generalizability	5
Duru et al. (2010)	62 African American women in 3 churches (Catholic, African Methodist Episcopal, Seventh-Day Adventist), 60 years of age or older	Randomized controlled trial, 8-week, 90-minute multi-component (scripture reading, prayer, goal setting, community resource guide, walking competition)	Intervention group increased weekly steps by 9,883 compared to controls 2,426 (p=0.02); systolic blood pressure decreased by 12.5 mmHg in intervention group and 1.5 mmHg in controls (p=0.007)	Self-selection bias, small sample size, uncertain generalizability	6
Garland et al. (2007)	60 majority female, diagnosed with cancer, 35–79 years of age	Observational study, mindfulness-based stress reduction (MBSR) 8-week, 90-minute sessions + one 3-hour weekend retreat vs. healing through creative arts (HA)-6 week, 120-minute sessions	Compared to HA group, MBSR had better improvement in measures of spirituality (p=0.029), anxiety (p=0.038), overall stress symptoms (p= 0.041), and disturbance (p=0.023)	Lack of randomization, small sample size, uncertain generalizability	9
Goldfinger et al. (2008)	26 overweight/obese African Americans, average age 68 years	Pre-post test, 10 week, 90-minute peer-led nutrition and exercise weight loss course	Post-intervention mean loss of 4.4 pounds at 10 weeks, 8.4 pounds at 22 weeks, and 9.8 pounds at 1 year	Lack of randomization, small sample size, uncertain generalizability	2
Holt et al. (2013)	16 churches, 285 adults, 60% female, 50–74 years of age	Randomized controlled trial, 2-group community advisor lay-led educational series on colorectal cancer screening with spiritual themes	Post-intervention FOBT increase in spiritually based group (2%) vs. nonspiritual group (9%) (p=0.0257)	Uncertain generalizability	5
Ka’opua et al. (2011)	198 women in six churches, 18–55 years of age	Randomized controlled breast cancer feasibility study, one 90-minute session, introduced by minister during worship service	Intervention improvements in awareness (χ2 = 6.82, p < 0.01) and intent to seek yearly mammograms (χ2 = 6.52, p < .05)	Self-reporting, uncertain generalizability	9
Kelly et al. (2011)	1726 outpatient adults, 18 years or older	Randomized controlled trial, 12 weekly sessions of cognitive behavioral therapy (CBT), motivational enhancement therapy, or 12-step facilitated therapy	At 3–15 month follow-up (outpatient and aftercare), 12-step facilitation therapy increased spiritual practices (outpatient, Beta= 0.14 p<0.0001; aftercare, beta=0.23, p<0.0001); proportion days abstinent (outpatient beta =0.21, p<0.0001; aftercare beta=0.24, p<0.0001); and drinks per drinking day (outpatient beta = −0.18, p<0.0001; aftercare beta = −0.25, p<0.0001)	Uncertain generalizability	9
Koenig et al. (2015)	132 non-psychotic chronic illness patients with depression, 18–55 years of age	Randomized controlled trial, 10-week, 50-minute sessions of religiously based cognitive behavioral therapy (RCBT) compared to cognitive behavioral therapy (CBT)	Adherence greater in RCBT vs. CBT (85.7% vs. 65.9%, p=0.10); no significant difference in outcomes between the two groups (B=0.33; SE, 1.80; p=0.86)	Small sample size, uncertain generalizability	9
Koszycki et al. (2014)	23 predominantly white women with generalized anxiety disorder, 18 years or older	Randomized controlled trial, 12-week, 50-minute sessions using multi-faith, spiritually based treatment	Compared to controls, spiritually-based intervention better outcomes for anxiety (F=13.57, p=0.001), illness severity, (F= 17.51, p < 0.001), self-report worry (F= 9.92, p= 0.005), intolerance of uncertainty (F= 11.93, p=0.003), and spiritual well-being (F= 12.31, p=0.002)	Social desirability bias, small sample size, uncertain generalizability	7
Langford et al. (2015)	745 African American adults from 16 churches, average age 51 years (treatment) and 45 years (controls)	Pre-post test, adapted Body & Soul program with pastoral support, church activities, church environment, and peer counseling	Enrollment higher in intervention group than in comparison group members (OR=1.94, 95% CI: 1.08–3.47, p=0.03)	Lack of randomization	4
Liu et al. (2008)	30 female breast cancer outpatients, 40–60 years of age	Randomized controlled trial, 10-week, 180-minute session, body-mind-spirit group therapy integrating Chinese Buddhism, Taoism, and Western treatment	Intervention group had better reduction in anxiety inventory than control group ((group · time interaction F (1, 24) = 5.51, p= 0.03)	Small sample size, uncertain generalizability	6
McCain et al. (2008)	387 HIV-infected men and women, average age 42.2 years	Randomized controlled trial, 10-week stress management approaches (cognitive-behavioral relaxation training (RLXN); focused Tai Chi training (TCHI); spiritual growth group (SPRT); or wait-listed control group (CTRL))	Cognitive relaxation and Tai Chi training groups used less emotion-focused coping than controls (p=0.030); all groups had augmented lymphocyte proliferative function (p=0.039)	Uncertain generalizability	6
Parker et al. (2010)	35 African American women from two churches, 25–64 years of age	Pre/post test, 10-week weight loss educational program incorporating scriptures, diet, and physical activity	Intervention led to significant reductions in weight, (Z=2.77, p<0.01), and improvements in systolic blood pressure (Z=− 1.97, p=0.05), body mass index (Z=−2.55, p=0.01), and physical activity (Z=−2.74, p<0.01)	Selection bias, small sample size, uncertain generalizability	5
Petry et al. (2008)	187 African American cocaine abusers and alcohol users, 18 years of age or older	Randomized controlled trial, 2–4 weeks of 3–4 sessions of contingency management (CM) treatment with/without engagement in religious activities	Religious activity engagers remained in treatment longer (F= 8.29, p<0.001), had longer abstinence durations (F = 9.42, p<0.01), and submitted more substance-negative samples (F=5.91, p <0.02) than non-engagers	Small sample size, uncertain generalizability	5
Rosmarin et al. (2010)	261 Jewish participants (Hassidic, Yeshiva Orthodox, Modern Orthodox, Conservative, Reform, other), 18 years of age or older	Randomized controlled trial, Internet spiritually based, 25–30 minute audio/video strategies (spiritually integrated treatment, SIT) for coping with stress and worry every 2 days for 2 weeks	Large improvements in stress (F(2,92) = 5.82, p < .005, n^2^ = 0.11); worry F(2,91) = 12.15, p < 0.001, n^2^ = .21); depression (F(2, 89) = 25.88, p< 0.001, n^2^ =0.23); and intolerance of uncertainty (F(2,87 =3.72, p < 0.05)); and greater belief in treatment credibility (t(116) = 2.7, p< 0.01)	Selection bias, small sample size, use of self-reported outcome measures, uncertain generalizability	10
Samuel-Hodge et al. (2009)	201 African Americans with type 2 diabetes from 34 churches, 20 years of age or older	Randomized controlled trial, 8-month special intervention (SI)-1 individual counseling, 12 group sessions, monthly phone contacts, and 3 postcards followed by 4-month monthly contacts compared to minimal intervention (MI)-mailed standard education	A1C post-intervention changes: special intervention participants (7.4%) and minimal intervention (7.8%) (95% confidence interval [CI] 0.1–0.6, p= 0.009)	Uncertain generalizability	2
Sattin et al. (2016)	604 overweight African Americans in 20 churches, 20–64 years of age	Cluster randomized controlled trial, Fit, Body and Soul (FBAS); 12-week 60-minute lay advisor led sessions vs. health education (HE)-information only	FBAS group more likely (13 %) than HE group (3 %) to achieve a 7 % weight loss (p=0.001) at 12 weeks; decline in fasting blood glucose (10.93 mg/dl) vs 4.22 mg/dl increase in HE group (p = 0.017), with larger differences at 12-months (intervention, 12.38 mg/dl decrease; HE group, 4.44 mg/dl increase) (p = 0.021)	Selection bias, uncertain generalizability	2
Studts et al. (2012)	345 White women in 29 churches, 40–64 years of age	Randomized controlled trial, lay health advisor home visits and newsletter to address barriers to cervical cancer screening	Treatment group (17.6% screened) had over twice the odds of wait-list controls (11.2% screened) of reporting Pap test receipt post-intervention, OR=2.56, 95% CI:1.03–6.38, p=0.04; recently screened (last Pap >1 but >5 years ago) had significantly higher odds of obtaining screening than rarely or never screened (last Pap ≥5 years ago), OR=2.50, 95%CI: 1.48–4.25, p=0.001	Small sample size, use of self-reported outcomes, uncertain generalizability	3
Tettey et al. (2017)	221 African Americans in 14 churches, 48–71 years of age	Pre-post test, faith-based cardiovascular health training program for lay health educators using a train- the-trainer model, integrated with biblical scripture, African American culture, and faith culture	Post-integration reductions in systolic BP (4.48 mmHg, p= 0.001), diastolic BP (3.38 mmHg, p=0.001), weight (3 lbs., p = 0.001), and BMI (0.46, p = 0.001); and improvements in cardiovascular disease health assessment scores (p=0.001)	Uncertain generalizability	5
Wachholtz et al. (2008)	83 migraine headache, medication naïve undergraduate students, average age 19 years old	Randomized control trial, 20-minute daily practice (spiritual meditation; internal secular meditation; external secular meditation; or relaxation only) ≥15 days	Intervention group had decreases in migraine headaches ((F(3,79) = 15.68, p < 0.001) and anxiety (F (3,79) = 3.31, p < 0.05); increases in pain tolerance (F(3,79) = 4.00, p < 0.01), daily spiritual experiences (F (3,79) = 2.67, p <0.05), and existential well being (F (3,79) = 2.13, p <0.05)	Small sample size, uncertain generalizability	5
Wilcox et al. (2007)	889 members from 303 African Methodist Episcopal churches and 571 members from 20 randomly selected churches	Randomized controlled trial (delayed group intervention) to increase physical activity (PA) among African Americans	Moderate intensity PA: intervention (88) vs. control (74), (p=0.003); met PA recommendations: intervention (31) vs. control (19) (p=0.02); stage of readiness for change: intervention (50) vs. control (33) (p=0.0006) and fruit and vegetable intake recommendations intervention (4.1) vs. control (3.1) (p<0.0001)	Selection bias	2
Williams et al. (2016)	225 low-income 3rd–5th graders, 8–11 years of age	Quasi-experimental design, engage children to reduce unhealthy eating and promote energy balance, 3 1-hour, 3 consecutive-day assembly-style sessions	Decline in purchased calories of 20% (p < 0.01) and unhealthy foods (p < 0.01)	Convenience sample, lack of randomization, uncertain generalizability	2
Wingood et al. (2013)	134 African American women, 18–34 years of age	Randomized controlled trial, 2 3-hour HIV prevention with faith components (abstinence, religious social capital, values of AA Christian women, religious coping)	Intervention effects on consistent condom use in the past 90 days and other sexual behaviors; (P4 3.57 (1.52, 8.39), p=0.004); number of weeks abstinent (2.4 times more likely to report > 30 days abstinent, p<0.001); significant increase condom-negotiation self-efficacy (mean difference = 2.36; p< 0.001)	Convenience sample, uncertain generalizability	10
